# Long-Term Efficacy of Disinfectants Against Ebola Virus and Nipah Virus for Use in Containment Level 4 Laboratories

**DOI:** 10.3390/v18030314

**Published:** 2026-03-03

**Authors:** Amanda L. Phelps, Lin Eastaugh, James S. Findlay, Ruth Thom, Mark S. Lever, Sophie J. Smither

**Affiliations:** Human and Biological Advantage, Defence Science and Technology Laboratory (Dstl), Porton Down, Salisbury SP4 0JQ, UK; alphelps@dstl.gov.uk (A.L.P.); lseastaugh@dstl.gov.uk (L.E.); jsfindlay@dstl.gov.uk (J.S.F.); reholloway@dstl.gov.uk (R.T.); mslever@dstl.gov.uk (M.S.L.)

**Keywords:** Ebola virus, Nipah virus, disinfectant, laboratory, Containment Level 4, Biosafety Level 4, efficacy, safety

## Abstract

Effective disinfectants are a requirement for safe operation within Containment Level 4 (CL4) laboratories. High-containment processes often demand that disinfectants remain effective over prolonged periods of time. Four candidate quaternary ammonium-based disinfectants were tested at room temperature for activity against Ebola virus and Nipah virus with a 5 min contact time when disinfectant was freshly prepared and again after disinfectants were aged for 26 weeks. The concentrations tested, based on manufacturer recommendations, were either 5% or 10%. Candidates that demonstrated long-term efficacy were also evaluated for viral breakthrough using reduced disinfectant concentration (1.25% or 2.5%) or reduced contact time (1 min). Breakthrough of Ebola virus was observed with one of the disinfectants using a reduced concentration compared to the manufacturer’s recommendation.

## 1. Introduction

**Figure 1:**
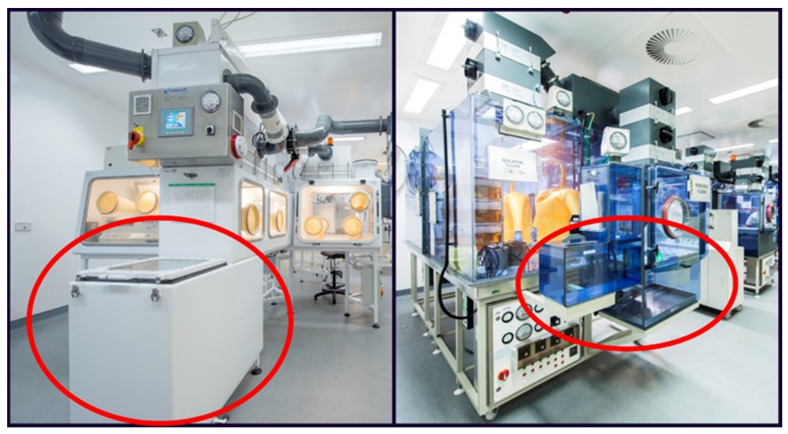
Large-volume dunk tanks. Dunk tanks (circled) are connected to a cabinet line (**left**) or rigid walled isolator (**right**) as a component of the primary containment system in a CL4 laboratory. Dunk tanks are filled with working-concentration disinfectant for a defined period of time (usually 4–6 months) whilst work is ongoing. Room dunk tanks to remove samples from the CL4 laboratory envelope are also present.

Effective disinfectants are a requirement for working in any microbiological environment, none more so than those laboratories at Containment Level 4 (CL4) (also called Biosafety Level 4 laboratories), where the highest-consequence viruses such as filoviruses, arenaviruses and henipaviruses are handled. In the UK, at Dstl, high-containment work is conducted within primary containment (in a Class III microbiological safety cabinet line or rigid walled isolator). Elsewhere, CL4 laboratories may use different systems (typically positive-pressure-suited systems). Disinfection is performed daily within a CL4 laboratory as part of safe working practice to reduce the titre of viable virus, for example by adding concentrated preparations to liquid waste in a final working concentration, or by using dilute disinfectant to reduce contamination on high-titre waste such as pipette tips or storage vials. At Dstl, liquid chemical disinfection is used in conjunction with formaldehyde fumigation, and sterilisation by autoclaving for waste generated during routine CL4 work. Disinfectants are also used in large-volume dunk tanks (Figure 1) and are an integral part of removing samples from the CL4 containment system and/or from the CL4 laboratories. The dunk tank from the primary containment systems to the room are used for removal of samples from the cabinet line or isolator, either for movement to connecting CL4 laboratories or for storage within the CL4 laboratory. The room dunk tanks are for removal of samples from the secondary containment area (the laboratory), for movement to non-connected laboratories, storage outside the laboratory, or for samples that are being moved to lower containment if using approved inactivation methods. Both dunk tanks are filled with the same concentration of the same disinfectant, and all samples leaving the laboratory would have gone through two rounds of immersion in dunk tanks. Due to the large volumes (>300 L per dunk tank) and the specialist waste disposal required for large volumes of disinfectant, it is impractical to change the disinfectant in dunk tanks daily or frequently, and therefore disinfectants with long-term efficacy that do not need to be prepared daily are essential. Disinfectants that remain active over long periods may also be useful for suited laboratories where they are frequently held in large volumes to be used for decontamination of positive pressure suits in a chemical shower system. Disinfectant with long-term efficacy may also be useful as part of emergency spill kits.

Identifying and understanding the efficacy profile of a selection of disinfectants against high-consequence viral pathogens provides flexibility and agility when operating under CL4 conditions. Commercial disinfectants undergo standard tests to meet national or international standards; however, they are not usually tested against the highest-consequence pathogens as the testing facilities are not CL4 laboratories. In this study, a selection of disinfectants were tested against two representative CL4 viruses, Ebola virus and Nipah virus, standardised with a 5 min contact time. Disinfectants were tested within 2 h of preparation (‘fresh’) and 26 weeks after preparation (‘aged’). The disinfectants were also tested for breakthrough at a reduced contact time of 1 min, and at concentrations of a quarter of the manufacturer-recommended value.

## 2. Materials and Methods

### 2.1. Disinfectants

Disinfectant selection was based on multiple criteria, including chemical safety and compatibility with extant laboratory and primary containment surfaces, availability in concentrate form, waste disposal considerations, and previously identified efficacy against RNA viruses. The availability of the disinfectant for purchase in the UK was also a key consideration. Three quaternary ammonium (QA)-based products were selected for evaluation. The extant disinfectant in use within Dstl CL4 labs, Desintex (Les Laboratoires Rochex, Juvigny, France) (https://www.laboratoires-rochex.com/produits/desintexbgf-desinfection-detergent-multiusage accessed on 20 February 2026), also a QA-based product, was utilised as a positive control alongside the three newly identified candidates:

• Chemgene MEDLAB (Byotrol, Warrington, UK)—recommended concentration 10%

https://byotrol.com/products/chemgene-medlab-multi-surface-disinfectant-concentrate-unfragranced/ (accessed on 20 February 2026)

• Surfanios Premium (Ecolab Bioquell, Andover, UK)—recommended concentration 5%

https://en-pl.ecolab.com/offerings/surfanios-premium (accessed on 20 February 2026)

• Selgiene Ultra (Selden Ltd., Buxton, UK)—recommended concentration 10%

https://www.selden.co.uk/products/product_information.asp?groupid=12&pil_id=624 (accessed on 20 February 2026)

### 2.2. Viruses and Cells

Ebola virus Mayinga (EBOV) was supplied by Battelle National Biodefense Institute (BNBI) under authorisation of the Center for Disease Control and Prevention (CDC, Atlanta, GA, USA). Nipah virus Malaysia (NiV) was provided by the Viral Special Pathogens Branch, CDC (Atlanta, GA, USA). Growth and enumeration of EBOV or NiV was performed in Vero C1008 cells (ECCAC Cat. No. 85020206) maintained in Dulbecco’s minimum essential tissue culture medium (TCM) (Sigma, Welwyn Garden City, UK) supplemented with 2% foetal calf serum, 1% L-glutamine, and 1% penicillin/streptomycin (all Sigma, Welwyn Garden City, UK). All incubations were for one week at 37 °C and 5% CO_2_. Enumeration of the virus was by 50% tissue culture infectious dose (TCID_50_) assay as described previously [1], where the lower limit of quantification is 10 TCID_50_/mL.

### 2.3. Disinfectant Efficacy Testing

**Figure 2:**
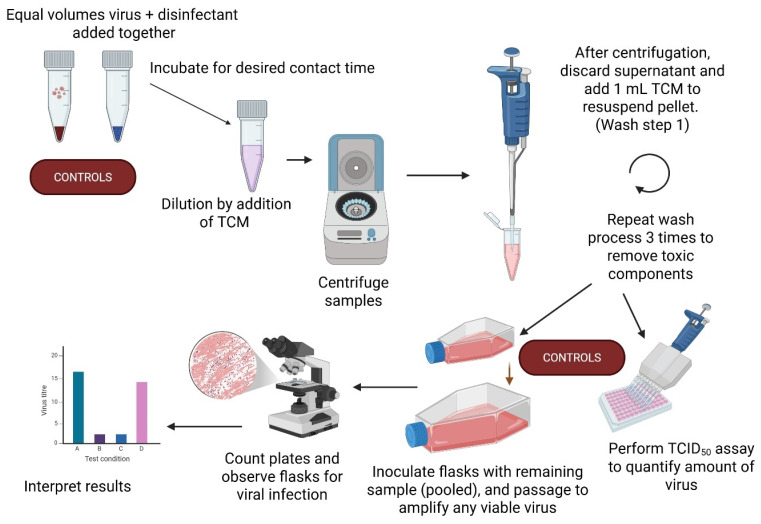
Schematic representation of the protocol used to test disinfectant efficacy against high-consequence viruses. The graph shown in ‘Interpret results’ is just a representation of this stage and is not showing any real data or results. Image created in BioRender. Sophie Smither 2025. https://app.biorender.com/illustrations/689f2c6d7430115aca133b00?slideId=123d7824-f41c-4f5f-ab33-b9483b761517, accessed on 20 February 2026.

The disinfectants were prepared in tap water at 2× the concentration to be tested so that when mixed 1:1 with virus they would be at the required manufacturer-recommended working concentration. The final concentrations, prepared to the manufacturer’s instructions, were 5% (*v*/*v*) for Surfanios Premium and Desintex, and 10% (*v*/*v*) for Chemgene MEDLAB and Selgiene Ultra. All disinfectants were stored at room temperature. A schematic of the testing method, whereby the entirety of the sample was tested, is shown in Figure 2; this method was as previously decribed [2]. The tap water used begins as hard water, with calcium amounts > 100 mg/L, but is softened by a standard water softening system. In the British Standards, the requirement for virucidal activity is a 4 log10 reduction (99.99 %) [3]

Equal volumes of the disinfectant and virus (50 µL) were added to a screw cap micro tube (Sarstedt, Nümbrecht, Germnay), in triplicate, for the desired contact time (5 min). The two droplets of disinfectant and virus were in direct contact but were not mixed. After 5 min contact time, 1 mL of TCM was added to provide a large-volume dilution of the disinfectant. The sample was then immediately centrifuged at 10K rpm for 5 min (MiniSpin microcentrifuge, Eppendorf, Stevenage, UK) and the supernatant discarded. A further 1 mL TCM was added to the tube, the sample resuspended, and the procedure repeated for a total of 3 washes (total of 3 centrifugation steps). An equal volume of virus and TCM were added together for 5 min in triplicate and treated as above as the positive control, to determine the maximum recovery of viable virus in the absence of any disinfectant. For toxicity control of the disinfectants, an equal volume of disinfectant and TCM (or TCM + TCM) was added together for 5 min, washed, and processed as for the test and controls, except that these samples were not performed in triplicate (no replicates within each round of testing, but performed in every round of testing). A standard TCID_50_ assay was performed on all samples to quantify the remaining viral load of washed samples. The remainder of all samples were pooled (or left as a single sample in the case of the toxicity controls) and inoculated into a T12.5 flask (Corning, NY, USA). After one week, the contents of all T12.5 flasks were further passaged into T25 flasks (Corning). Each serial passage was microscopically observed and scored for the presence or absence of viable virus (CPE), providing a qualitative output of infection.

### 2.4. Evaluation of Efficacy over Time and Breakthrough Testing

The efficacy testing protocol described above was used to test disinfectants that had been freshly prepared on the day of testing, and then those same disinfectants after being left to age at room temperature for 6 months to represent disinfectant aged in dunk tanks. Testing was done with EBOV and NiV at both time-points. Fresh and aged efficacy testing was performed with disinfectants at the manufacturer-recommended concentrations.

In addition, to test breakthrough points, reduced concentrations (a quarter of the manufacturer recommendation) were tested. Reduced concentrations were made by diluting the already diluted disinfectants 1:4 in tap water to render a test concentration of 2.5% for Chemgene and Selgiene and 1.25% for Desintex and Surfanios. The reduced concentrations were tested for 5 min contact time. Reduced contact time (1 min) was also investigated using disinfectants at the original manufacturer-recommended concentration. The breakthrough testing was performed using the same method as described in Figure 2 and with EBOV and NiV. By the time of the breakthrough testing, the disinfectants were aged 16 months from the time of original preparation.

## 3. Results

### 3.1. Disinfectant Efficacy

The results of the quantitative disinfectant testing with freshly prepared and 26-week-aged disinfectants for 5 min contact time are shown in Table 1. The mean titre of virus recovered from samples treated with different disinfectants is shown. No virus was detected, by TCID_50_ assay, in any of the four disinfectant test samples, for either the freshly prepared or aged disinfectant. The mean recovery of EBOV from positive controls was 1.19 × 10^4^ (1 × 10^4^–1.58 × 10^4^) TCID_50_/mL, and 5.13 × 10^4^ (4.68 × 10^4^–5.62 × 10^4^) TCID_50_/mL for NiV in assays assessing freshly prepared disinfectant. In assays assessing aged disinfectant, the mean recovery of EBOV from positive controls was 2.81 × 10^4^ (5.62 × 10^3^–4.7 × 10^4^) TCID_50_/mL, and 5.62 × 10^4^ (single replicate available in this assay) TCID_50_/mL for NiV.

Blind serial passage of samples supports the findings of the TCID_50_ assays in that, after a total of 14 days’ incubation (2× passage), no CPE (or toxicity) was observed in any of the four disinfectant test samples, for either freshly prepared or aged disinfectant. Both the EBOV and NiV positive control flasks were scored as positive for CPE, and the TCM only control flasks were also scored as negative for CPE.

The combination of both quantitative (TCID_50_/mL) and qualitative (serial passage in flasks) assays utilises 100% of the sample. Additionally, serial passage in flasks provides an opportunity for samples that may contain ≤10 TCID_50_/mL to be amplified and replicate, producing observable CPE. Samples negative for CPE after two passages in flasks (and negative by TCID_50_ assay) were considered to have successfully disinfected all available viable virus. An overall >4 Log10 reduction in viral titre was achieved for both EBOV and NiV when treated with freshly prepared and aged disinfectant, with a 5 min contact time.

### 3.2. Breakthrough Testing with Reduced Disinfectant Contact Time

Tests were carried out with the same concentrations of disinfectant as listed in Table 1, but with 1 min contact time against EBOV or NiV, at room temperature, using 16-month-aged disinfectant. The mean recovery from positive controls was 1.23 × 10^4^ (1 × 10^4^–1.7 × 10^4^) TCID_50_/mL [4.1 Log10] for EBOV, and 1.52 × 10^4^ (1.31 × 10^4^–1.95 × 10^4^) TCID_50_/mL [4.2 Log10] for NiV. As with the 5 min contact time, no virus was recovered by TCID_50_ assay, from any of the four disinfectant test samples, and no CPE was observed in blind passage, except for the virus positive controls. A >4 Log10 reduction in viral titre was achieved for both EBOV and NiV when treated with the significantly aged disinfectant, with a 1 min contact time. A lack of breakthrough with 1 min contact provides a significant safety margin for the proposed routine use of a minimum of 5 min contact time within the Dstl laboratory setting.

### 3.3. Breakthrough Testing with Reduced Disinfectant Concentration

**Figure 3:**
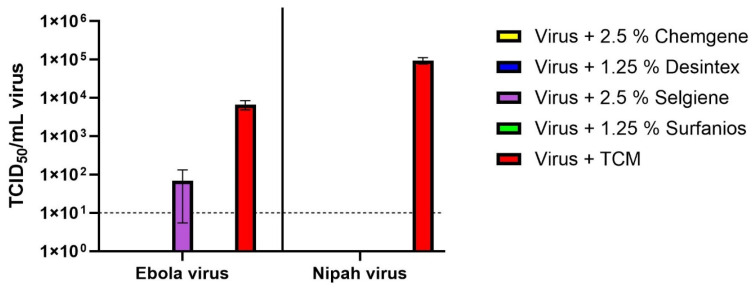
Disinfectant concentration breakthrough testing. Mean titre of virus (TCID_50_/mL) recovered after testing four disinfectants at reduced concentration against Nipah virus or Ebola virus with a 5 min contact time is shown. The error bars are the SEM from three replicates where any virus was recovered. No coloured bar = no virus recovered from those conditions. The dotted line at 10^1^ is the limit of quantification of the TCID_50_ assay. TCID_50_: 50% tissue culture infectious dose.

The disinfectants (now aged 16 months) were further diluted 1:4 in tap water to understand whether virus breakthrough would occur with reduced disinfectant concentrations (Figure 3). The working concentrations therefore were 2.5% for Chemgene and Selgiene, and 1.25% for Surfanios and Desintex, with a standard contact time of 5 min.

The mean recovery from positive controls was 6.66 × 10^3^ (3.73 × 10^3^–1 × 10^4^) TCID_50_/mL [3.8 Log10] for EBOV, and 9.54 × 10^4^ (7.63 × 10^4^–1.31 × 10^5^) TCID_50_/mL [5.0 Log10] for NiV. As with the 5–10% concentrations of disinfectant, no NiV virus was recovered by TCID_50_ assay from any of the four disinfectant test samples, and no CPE was observed in blind passage, except for the virus positive control.

The same is true for EBOV, with the exception of 2.5% Selgiene, where a mean titre of 69 (<10–195) TCID_50_/mL [2.0 Log10] was recovered. Two of the three EBOV + 2.5% Selgiene samples were below the limit of quantification of the TCID_50_ assay (10), but CPE could still be observed in some of the wells in the column containing the neat sample. The third replicate had a quantifiable titre of 195 TCID_50_/mL. Blind passage of these Selgiene-treated samples was also positive for EBOV. A parallel control treated with 10% Selgiene aligned with the results described earlier; no viable virus was recovered by TCID_50_ assay or blind passage. These data provide evidence of EBOV breakthrough at much-reduced concentrations of 16-month-aged Selgiene disinfectant.

Three of the 16-month-aged disinfectants provided a ~4 Log10 reduction in titre when diluted 1:4 from that recommended by the respective manufacturers, providing an additional safety margin for the proposed routine use of a minimum of 5% for Chemgene and Selgiene, and 10% for Surfanios.

## 4. Discussion

Efficacy testing of disinfectants has been carried out previously against EBOV [2,4,5,6,7] and NiV [7,8], typically in order to observe the immediate virucidal effect rather than long-term efficacy. Similarly, standard testing of disinfectants by accredited testing facilities does not assess disinfectant age, nor are assessments performed with high-consequence pathogens requiring CL4 containment facilities. The working processes in our laboratory require empirical evidence of both disinfectant efficacy and disinfectant longevity against high-hazard pathogens such as EBOV and NiV. Here, we selected three quaternary ammonium (QA)-based products based on our internally driven criteria, to understand the efficacy profile of a selection of disinfectants against high-consequence viral pathogens to provide flexibility and agility when operating under CL4 conditions. A wide range of disinfectant types were initially considered, including aldehyde-, alcohol-, and acid-based products, but ultimately down-selection resulted in the testing of QA-based products only in this study. It is quite possible, however, that other products may demonstrate similar efficacy. All potential products therefore should be considered individually based on efficacy, context of use, and safety. Quaternary ammonium compounds are a common group of disinfectants, the most widely used of which for Biosafety Level 4 laboratory chemical showers in the U.S., Australia, and Asia is Micro-Chem Plus, another QA compound. Although Micro-Chem has undergone validation in Canada [9], it is not available in Europe, and thus not an option for us. Micro-Chem is typically used at 5% and for contact times varying from 3 min to 20 min depending on an individual institution’s protocols (BSL4ZNET members, personal communication), presenting similar parameters to those of the disinfectants we have tested. Our data could be considered as alternative options for chemical showers with long-term efficacy demonstrated. Further evaluation would be needed for testing in the context of a chemical shower for positive-pressure-suited systems.

Our data provide evidence of robust disinfection efficacy for three QA-based products, used at the manufacturer-recommended concentrations (5 or 10%) and for a 5 min contact time. Moreover, the data have also shown that disinfectant efficacy was maintained up to 16 months with a significantly reduced contact time (1 min) and, for three of the four disinfectants, a reduced concentration (1:4 dilution). This provides substantial confidence and safety redundancy for the proposed laboratory uses at Dstl.

These assessments were made using just two viruses, both enveloped RNA viruses. Whilst all current viruses that are handled at CL4 (at Dstl) fall into this category, testing should be extended to DNA viruses and/or non-enveloped viruses if the disinfectant is to be considered for wider usage. Manufacturers of these products claim efficacy against more robust targets (e.g., polio, norovirus) and hence it is likely, although unproven, that the disinfectants would have efficacy against other viruses not currently tested.

It is worth noting that our starting virus stocks were at the highest concentration available (>10^6^), but due to the methodologies employed (using small volumes of virus, followed by diluting by washing), the maximum overall recoverable viral load was ~10^4^ TCID_50_/mL. Higher titres might be encountered in typical laboratory usage. Disinfection, however, is defined as the reduction in titre and not the complete removal of viable material (which is the definition of sterilisation), and should always be considered alongside other control measures and working practices for safe handling and working. Our testing, wherein we held virus and disinfectant at a ratio of 1:1 (without mixing), might also be considered representative of a worst-case scenario; in reality, the ratio of disinfectant is likely to be much greater than that of virus when used for either surface decontamination or for action taken against a spill. The same disinfectant is used in the CL4 laboratory for disinfection of all sample types that are to be disposed of; there is no differentiation between in vitro and in vivo waste in terms of the disinfection used. Where samples are to be inactivated for removal to be handled at lower containment, e.g., animal tissues via formalin fixation, other chemicals and in-house tested and validated methods are used.

Our testing method uses 100% of each sample and tests for viable virus through cell culture methods. This protocol has proved effective for all viruses handled at CL4 to date; however, it might not work for viruses that do not grow well or produce clear cytopathic effects in cells. In that case, other quantification methods would have to be considered. The use of RT-qPCR would have to be interpreted carefully, as it is not a measurement of viable virus only. Reporter cell lines that indicate whether a cell has been infected might be an alternative option.

It is also important to point out that Dstl is not an accredited testing facility, and these experiments were not conducted according to any recognised standard. These data are shared without commitment or prejudice, for the purposes of scientific investigation and internal validation of procedures.

© Crown copyright (2026), Dstl. This information is licensed under the Open Government Licence v3.0. To view this licence, visit https://www.nationalarchives.gov.uk/doc/open-government-licence/, accessed on 20 February 2026.

## Figures and Tables

**Table 1:** Mean titre of virus in (TCID_50_/mL) recovered from samples treated with different disinfectants for 5 min contact time. The Log10 titre is given in [square brackets]. TCID_50_: 50% tissue culture infectious dose.

Sample	Freshly Prepared Disinfectant		26-Week-Aged Disinfectant	
	Ebola Virus	Nipah Virus	Ebola Virus	Nipah Virus
Virus + TCM	1.19 × 10^4^ [4.1]	5.13 × 10^4^ [4.7]	2.81 × 10^4^ [4.4]	5.62 × 10^4^ [4.7]
Virus + 10% Chemgene	0	0	0	0
Virus + 5% Desintex	0	0	0	0
Virus + 10% Selgiene	0	0	0	0
Virus + 5% Surfanios	0	0	0	0

## Data Availability

Data are contained within the article. Further inquiries can be directed to the corresponding author.

## References

[1] Smither S.J., Eastaugh L.S., Findlay J.S., O’Brien L.M., Thom R., Lever M.S. (2019). Survival and persistence of Nipah virus in blood and tissue culture media. Emerg. Microbes Infect..

[2] Smither S., Phelps A., Eastaugh L., Ngugi S., O’Brien L., Dutch A., Lever M.S. (2016). Effectiveness of Four Disinfectants against Ebola Virus on Different Materials. Viruses.

[3] (2018). Chemical Disinfectants and Antiseptics. Quantitative Non-Porous Surface Test Without Mechanical Action for the Evaluation of Virucidal Activity of Chemical Disinfectants Used in the Medical Area. Test Method and Requirements (Phase 2/Step 2).

[4] Cook B.W.M., Cutts T.A., Nikiforuk A.M., Leung A., Kobasa D., Theriault S.S. (2016). The Disinfection Characteristics of Ebola Virus Outbreak Variants. Sci. Rep..

[5] Huang Y., Xiao S., Song D., Yuan Z. (2022). Efficacy of disinfectants for inactivation of Ebola virus in suspension by integrated cell culture coupled with real-time RT–PCR. J. Hosp. Infect..

[6] Jonsdottir H.R., Zysset D., Lenz N., Siegrist D., Ruedin Y., Ryter S., Züst R., Geissmann Y., Ackermann-Gäumann R., Engler O.B. (2023). Virucidal activity of three standard chemical disinfectants against Ebola virus suspended in tripartite soil and whole blood. Sci. Rep..

[7] Huang Y., Xiao S., Song D., Yuan Z. (2022). Evaluation and comparison of three virucidal agents on inactivation of Nipah virus. Sci. Rep..

[8] Smither S.J., Eastaugh L.S., O’Brien L.M., Phelps A.L., Lever M.S. (2022). Aerosol Survival, Disinfection and Formalin Inactivation of Nipah Virus. Viruses.

[9] Leung A., Cutts T., Krishnan J. (2024). Decontamination Validation of the BSL-4 Chemical Disinfectant Deluge Shower System. Appl. Biosaf..

